# Improved drowsiness detection in drivers through optimum pairing of EEG features using an optimal EEG channel comparable to a multichannel EEG system

**DOI:** 10.1007/s11517-025-03375-1

**Published:** 2025-05-16

**Authors:** Riaz Minhas, Nur Yasin Peker, Mustafa Abdullah Hakkoz, Semih Arbatli, Yeliz Celik, Cigdem Eroglu Erdem, Yuksel Peker, Beren Semiz

**Affiliations:** 1 College of Engineering, Koc University, 34450 Istanbul, Turkey; 2 Department of Mechatronics Engineering, Sakarya University of Applied Sciences, 54050 Sakarya, Turkey; 3 Graduate School of Computer Engineering, Istanbul Technical University, 34469 Istanbul, Turkey; 4 Graduate School of Health Sciences, Koc University, 34010 Istanbul, Turkey; 5 Research Center for Translational Medicine (KUTTAM), Koc University, 34010 Istanbul, Turkey; 6 Department of Electrical and Electronics Engineering, Ozyegin University, 34794 Istanbul, Turkey; 7 Department of Pulmonary Medicine, School of Medicine, Koc University, 34010 Istanbul, Turkey; 8 University of Gothenburg, Lund University, Sweden and University of Pittsburgh, Sweden, PA USA

**Keywords:** Drivers, Drowsiness detection, Electroencephalography, Multichannel EEG system, Optimal pairing of EEG features, Optimal EEG channel

## Abstract

Multichannel electroencephalography (EEG)-based drowsiness detection (DD) offers higher coverage but comes with increased computational demands, hardware requirements, and user discomfort, whereas single-channel devices are cost-effective and user-friendly but provide lower coverage. We hypothesized that an optimal channel with optimum paired EEG features could achieve coverage comparable to a multichannel system. Subject-specific, EEG-feature-specific thresholding techniques were introduced to classify 927 EEG epochs, derived from visual-based scoring through image processing of fifty drivers’ facial expressions during a 50-min driving simulation, using six individual EEG channels with paired features. Ten normalized EEG features were extracted per epoch using discrete wavelet transform (DWT), and seven thresholding techniques were applied to identify the most consistent method across subjects. Epochs were classified as drowsy or wakeful based on whether their normalized values exceeded or fell below a specific threshold. We then assessed the coverage of each channel by comparing EEG patterns with visual-based scoring. To determine the optimal feature pair for classifying each epoch in alignment with visual-based scoring, 45 feature combinations were evaluated. The pairing of power spectral density (PSD) alpha and PSD theta in channels Frontal4 (F4) and Occipital2 (O2) yielded the highest coverage, achieving 96.1% and 95% with corresponding accuracies of 95.4% and 94.7%, respectively. These results slightly surpassed the coverage achieved using six channels with a single feature, with increases of 1.47% for F4 and 0.32% for O2. Our study demonstrates that an optimal EEG channel with optimum paired EEG features can reduce channels from six to one, lowering computational demands for wearable DD devices.

**Graphical abstract:**

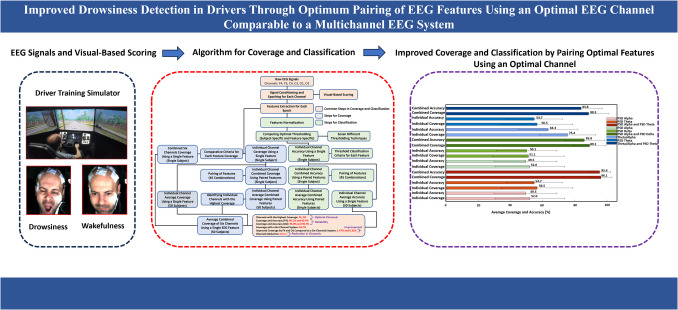

## Introduction

Drowsiness is characterized by the transition from wakefulness to sleep, accompanied by a noticeable decline in cognitive and psychomotor functions, resulting in increased reaction times [1, 2]. These drowsy episodes frequently arise during monotonous yet mentally demanding tasks, compromising efficacy and performance, particularly evident during driving, thereby increasing the risk of road accidents [3]. A meta-analysis revealed a significant association between drowsy driving and crash involvement (odds ratios (OR) = 1.29, 95% confidence interval (CI) 1.24–1.34) [4]. Furthermore, the onset of drowsiness has previously resulted in catastrophic accidents, leading to over 200 fatalities and more than 400 injuries in a single incident [5]. According to the American Automobile Association (AAA), drowsy driving contributes to one-sixth of fatal traffic accidents and one-eighth of accidents requiring hospitalization for drivers or passengers [6].

Previous approaches to DD systems in simulated driving environments primarily use three measurement approaches: (1) vehicle-based measures (such as lane deviation, steering entropy, and out-of-road events) [7, 8], (2) behavioral measures including eye movement [9, 10], yawning [11], and head-nodding [12], and (3) physiological signal measures, encompassing electrocardiogram (ECG), electromyogram (EMG), electrooculogram (EOG), and EEG [13–15]. Among these, EEG is widely recognized as the most direct and sensitive measure for detecting drowsiness onset because it captures real-time neural activity related to cognitive and alertness states. Studies have demonstrated that EEG-based features, particularly those derived from power spectral analysis, provide earlier and more reliable indicators of drowsiness compared to behavioral and vehicle-based metrics, which often manifest after drowsiness has already impaired driving performance [16, 17]. In contrast, behavioral measures can be influenced by external factors (e.g., lighting conditions, head position), and vehicle-based measures typically detect drowsiness only after performance deteriorates. Thus, EEG offers a superior combination of timeliness and accuracy, making it the preferred modality for drowsiness detection in high-risk scenarios such as driving. EEG works by recording the brain’s electrical activity through electrodes placed on the scalp, with drowsiness-related activity primarily occurring in four key frequency bands: beta (15–30 Hz), alpha (7.5–15 Hz), theta (4–7.5 Hz), and delta (1–4 Hz) [18].

Although EEG systems designed for research and medical use often employ multichannel setups, such as the use of at least four channels in the Multiple Sleep Latency Test (MSLT) and Maintenance of Wakefulness Test (MWT) to enhance coverage—their complex assembly and prolonged application times can lead to user discomfort, making them impractical for real-world applications [19, 20]. Conversely, low-cost EEG systems are quicker to install and user-friendly, but their fewer electrodes (typically up to 2) often result in lower coverage [21–28]. Given this trade-off, reducing the number of EEG channels to a single channel, by identifying the most effective EEG channel that maintains high coverage comparable to a multi-channel system while using a minimal number of EEG features, is crucial for addressing real-world constraints in DD. In this study, this channel is referred to as the “optimal channel,” denoting the best-performing channel among those analyzed. The coverage of a channel refers to its ability to capture variations in EEG patterns during epochs consistent with the reference system.

### Related works

Previous research has effectively identified drowsiness using a minimal number of EEG channels and features (as summarized in Table 1), albeit with limitations. Lin et al. [21] used subjects’ response times to lane departure events as ground truth to develop a system based on a unipolar single EEG channel. This system utilized θ and α-based features from the occipital area (Oz) and achieved an accuracy of 82.8%. Punsawad et al. [22] employed drivers’ self-assessment, where they pressed a button when feeling drowsy, to develop a system based on bipolar EEG channels. This system applied weighted θ∼β features from the temporal and parietal areas (T3 and P4 or T4 and P3), achieving 90.4% accuracy. Using the Rechtschaffen and Kales (R&K) sleep scoring standard as a reference, Hal BV et al. [23] developed a unipolar single-channel EEG-based system and extracted α and β features from the frontal area (Fp1), achieving 81% accuracy. Furthermore, Li et al. [24] employed the percentage of eyelid closure over time (PERCLOS) as a reference, where a support vector machine-based posterior probabilistic model (SVMPPM) achieved 91.92% accuracy by utilizing channels O1 and O2, along with FFT-based theta (θ), alpha (α), and beta (β) features. Ogino et al. [25] used a prefrontal (FP1) single-channel EEG device along with FFT-based theta (θ) and alpha (α) features, achieving a 72.7% classification accuracy using PSD with stepwise linear discriminant analysis (SWLDA) and support vector machine (SVM) models. Additionally, Shalash et al. [26] transformed the filtered EEG signal into a two-dimensional (2D) spectrogram, then classified it using AlexNet with transfer learning. FP1 and T3 channels were identified as the most accurate, achieving up to 90% and 91% accuracy, respectively, in detecting drowsiness states. Correa et al. [27] utilized a single bipolar EEG channel (C3-O1) along with seven features—central frequency, standard deviation, first quartile frequency, maximum frequency, IEEGθ, IEEGα, and asymmetry coefficient—to distinguish between alertness and sleepiness using a neural network classifier, achieving an average accuracy of 85.5%. Belakhdar et al. [28] also employed a single bipolar EEG channel (C3-O1) along with four features— FFT-based average power of [3 − 4] Hz, [3 − 4] Hz/alpha, theta/alpha, and mean power ratio in individual alpha frequency (IAF)—to detect drowsiness using a multi-layer perceptron (MLP) neural network, reaching an accuracy of 85.5%.

**Table 1:** This table presents a quantitative comparison between our study and previous state-of-the-art EEG-based drowsiness detection methods. Each study utilized minimal EEG channels and selected EEG features to achieve drowsiness classification. Our study demonstrates a higher accuracy (95.4%) using a single optimal channel (F4) with an optimal feature pair, reducing the need for multiple EEG channels while maintaining performance

Study	Reference system	EEG channel(s)	EEG feature(s)	Channel’s accuracy and methodology
Lin et al. [21]	Subjects’ response times to lane departure events	Single Channel: Oz	Two Features: FFT-based theta (θ) and alpha (α)	82.8% (Threshold-based classification)
Punsawad et al. [22]	Drivers'self-assessment (button press for drowsiness	Two Channels: T3 and P4 or T4 and P3	Two Features: FFT-based theta (θ) and beta (β)	90.4% (Threshold-based classification)
Hal et al. [23]	Rechtschaffen and Kales (R&K) sleep scoring	Single Channel: FP1	Two Features: FFT-based alpha (α) and beta (β)	81% (Threshold-based classification)
Li et al. [24]	PERCLOS	Two Channels: O1 and O2	Three Features: FFT-based theta (θ), alpha (α), and beta (β)	91.92% (Machine-learning-based classification)
Ogino et al. [25]	Karolinska Sleepiness Scale (KSS)	Single Channel: FP1	Two Features: FFT-based theta (θ) and alpha (α)	72.7% (Machine-learning-based classification)
Shalash et al. [26]	Not included a reference system	Single Channel: T3	Not reported	91% (Deep-learning-based classification)
Correa et al. [27]	Rechtschaffen and Kales (R&K) sleep scoring	Single-Differential Channel: C3-O1	Seven Features: Central frequency, standard deviation, first quartile frequency, maximum frequency, IEEGθ, IEEGα, and asymmetry coefficient	85.5% (Deep-learning-based classification)
Belakhdar et al. [28]	Rechtschaffen and Kales (R&K) sleep scoring	Single-Differential Channel: C3-O1	Four Features: FFT-based average power of [3 − 4]Hz, [3 − 4]Hz/alpha, theta/alpha, and mean power ratio in individual alpha frequency (IAF)	88.80% (Deep-learning-based classification)
**Present study**	**PERCLOS with associated limitations mitigated**	**Single optimal channel: F4**	**Optimal paired features: DWT-based PSD alpha (α) and PSD theta (θ)**	**95.4% (threshold-based classification)**

### Limitations in previous studies and a proposed solution

Lin et al. [21] overlooked potential delays between vehicle-generated alerts and EEG signals, as vehicle-based warnings typically occur later in the initial phase of drowsiness [17]. Punsawad et al. [22] and Ogino et al. [25] relied on drivers’ self-assessment for reference drowsiness, introducing subjective biases. Hal et al. [23], Correa et al. [27], and Belakhdar et al. [28] used manual sleep scoring based on the R&K standard, which is challenging, prone to error, and labor-intensive [29]. Additionally, its effectiveness may be compromised by epochs shorter than three seconds [30]. Li et al. [24] employed a personal computer-based driving simulator, which may not effectively mimic real-world driving conditions [31]. Shalash et al. [26] did not include a comparative analysis between their methodology and other established techniques or scoring systems for validation. Although the aforementioned research aimed to enhance the accuracy of drowsiness detection using 1 or 2 selected channels, chosen based on findings from previous studies, none attempted to systematically reduce the number of EEG channels by identifying the optimal channel through coverage analysis of the different channels analyzed. Additionally, they did not compare their minimal-channel system with a multi-channel system to evaluate performance trade-offs.

The present study aims to use visual-based scoring as a reference system to identify wakeful and drowsy episodes by integrating two metrics: PERCLOS and eyelid closure duration (CLOSDUR). Building on authors’ previous research [18, 32], which showed that the sensitivity in correlating EEG patterns with the episodes of visual-based scoring varies with channel number, location, and EEG feature type, this study focuses on reducing EEG channels while enhancing individual channel coverage. This is done by identifying the most effective paired EEG features for each channel and selecting the channel with the highest coverage. The coverage of this channel is then compared to a six-channel system to find an optimal channel with comparable or better performance. Additionally, each EEG epoch is classified as wakeful or drowsy by comparing the normalized EEG feature value to an optimal threshold, ensuring consistency with visual-based scoring (referred to as channel’s accuracy). To the authors’ knowledge, this is the first study comparing single-channel coverage to multichannel systems, identifying F4 as slightly more sensitive than O2 for detecting drowsiness. The major contributions of this paper are as follows:

1. The authors propose subject-specific and EEG feature-specific classification using optimal thresholding selected from seven techniques to achieve consistent results across all features and subjects (Sections. 2.2.2 and 3.3)
2. The study evaluates 45 combinations of EEG feature pairs to identify the optimal pair for classifying epochs as drowsy or wakeful, aligning with visual-based scoring, and determining coverage in matching EEG patterns with this scoring (Sections. 2.2.4 and 3.1)
3. For each channel, the authors compute the average coverage and accuracy for each feature within a pair, and also determine these metrics for the combined pair, across fifty subjects. The similarity between average coverage and average accuracy is believed to showcase the reliability of the methodology (Sections. 2.2.3–2.2.5 and 3.1)
4. Additionally, the study presents the enhancement in average coverage of the individual channels by optimal paired EEG features and compares their coverages with a six-channel system to identify the optimal channel (Sections. 2.2.6 and 3.2)

## Methods

### Datasets

In the current study, the authors utilized datasets from their previous research [18], where the high-fidelity XBUS PRO Driver Training Simulator (DTS), developed by ANGRUP Co. (Istanbul, Turkey), was employed. The DTS included a driver cabin, a camera system, a voice communication setup, acceleration and brake pedals, and steering controls. It offered both automatic and manual transmission modes and provided a variety of training scenarios. External devices, including a 6-channel EEG device with a sampling rate of 200 Hz and a camera capturing the driver’s facial expressions at 30 frames per second (fps), were seamlessly integrated with the simulator. A cohort of 50 professional drivers diagnosed with obstructive sleep apnea (OSA) with an Apnea–Hypopnea Index (AHI) of ≥ 5.0 events/h [33] participated in a 50-min simulated driving session conducted on a two-way highway with low traffic density, not exceeding a maximum allowable speed of 62 mph (80 km/h). All participants, who were free of acute illness, provided written informed consent. Additionally, they were instructed to abstain from consuming any stimulants or caffeinated beverages, including energy drinks and coffee, for 24 h before the experiment. Participants meeting eligibility criteria underwent the simulated driving session, which was scheduled between 08:00 a.m. and 10:00 a.m. The interior lights of the cabin were kept off to mimic real-world driving conditions.

The demographic characteristics of the participants were as follows: All were males, with ages ranging from 32 to 68 years (mean age, 47.9 ± 7.6 years). Their Body Mass Index (BMI) ranged from 23.5 to 41.9 kg/m^2^ (mean, 31.3 ± 4.4 kg/m^2^). Reported sleep duration from the previous night varied between 1 to 11 h, with an average of 6.3 ± 1.8 h. The Apnea–Hypopnea Index (AHI) ranged from 5 to 103.5 events per hour (mean, 29.8 ± 23.2 events per hour), and the Oxygen Desaturation Index (ODI) ranged from 1.0 to 87.8 events per hour (mean, 24.4 ± 22.7 events per hour) recorded during the previous night’s polysomnography. The EEG signals were acquired from all participants using electrodes placed on specific locations on the scalp according to the 10–20 system [34]: Frontal (F4 and F3), Central (C4 and C3), Occipital (O1 and O2), and two reference electrodes (M1 and M2) placed on the mastoids behind both ears. Based on the experimental setup, the EEG configuration was unipolar (referential) since each channel was recorded against a common reference electrode.

#### Visual-based scoring dataset

**Fig. 1:**
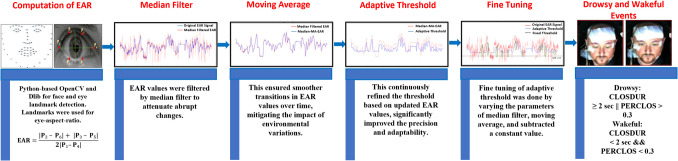
Steps for visual-based scoring: Step 1 extracts EAR values from video frames, step 2 applies a median filter to the EAR values, step 3 uses a moving average filter to smooth the signal, step 4 employs an adaptive threshold to enhance accuracy and adapt to signal conditions, step 5 fine-tunes the parameters and identifies consecutive signals falling below the threshold to detect blinks, and step 6 computes two metrics to confirm drowsy and wakeful episodes: PERCLOS, the ratio of frames with closed eyes to total frames, and CLOSDUR. The start and end times of these alternating episodes are saved in CSV files subject-wise, with labeling 1 for potential wakeful (odd rows) and 0 for potential drowsy (even rows) episodes

Visual-based scoring was derived from the integration of two metrics: PERCLOS and CLOSDUR, both widely acknowledged techniques for detecting drowsy and wakeful episodes by analyzing facial video recordings of drivers during simulated driving [35]. This dataset included timestamps of drowsy (*n* = 453) and wakeful (*n* = 474) episodes determined across fifty subjects. The complete methodology for calculating these events is explained in Fig. 1.

#### EEG dataset

The EEG signals, recorded for 50 min from each participant, including start and end times from all six channels, were stored in the European Data Format (EDF) using Noxturnal software [36]. Finite impulse response (FIR) filters were utilized, including both high-pass and low-pass filters, designed with an order of 25 using the equiripple design approach [18]. This choice was made to achieve sharp transition zones between the passband and stopband, crucial for accurate EEG signal analysis. The equiripple design method ensures minimal ripple in both the passband and stopband, providing optimal attenuation of unwanted frequencies while preserving the essential characteristics of the EEG signals. The high-pass filter, set at a cutoff frequency of 1 Hz, effectively attenuated low-frequency artifacts associated with eye blinks [37]. Conversely, the low-pass filter, operating at a cutoff frequency of 30 Hz, aimed to eliminate high-frequency noise and potential artifacts from EMG signals [38]. The filtered EEG data were then segmented into epochs corresponding to the timestamps of drowsy (*n* = 453) and wakeful (*n* = 474) episodes, as derived from visual-based scoring for each subject.

Subsequently, for each epoch, DWT was applied using the “db2” wavelet at level 3 decomposition. DWT effectively analyzes non-stationary EEG signals by providing high resolution in both time and frequency domains. This precision in time–frequency localization is essential for identifying specific EEG patterns related to brain activity [18, 39]. The “db2” wavelet was chosen due to its superior performance in classifying EEG signals, achieving high accuracy in distinguishing brain states [40, 41]. It has been shown to outperform other wavelets, such as coeif4, sym10, db1, and db6, in terms of classification accuracy [41]. The 3-level decomposition allows us to focus on critical frequency bands—delta (1–4 Hz), theta (4–7.5 Hz), alpha (7.5–15 Hz), and and beta (15–30 Hz)—by eliminating irrelevant frequency components and enhancing signal-to-noise ratio [42]. Ten specific EEG features were then computed for each epoch: PSD alpha, PSD theta, PSD delta, their ratios (theta/alpha, delta/alpha, and delta/theta), spectral entropy, spectral spread, spectral centroid, and spectral rolloff. This methodology was repeated for all channels across fifty subjects.

### Reducing EEG channels by ıdentifying the optimal channel using optimal paired EEG features

The authors’ previous study [32] assessed the sensitivity of individual EEG channels and different brain regions (frontal, central, occipital), as well as the collective sensitivity of all six channels, focusing on theta/alpha ratio. “Sensitivity” refers to a channel’s effectiveness in capturing variations in the EEG patterns corresponding to wakeful and drowsy states identified through vehicle-based scoring (on-road and off-road episodes) using a comparative criterion. This criterion compares the theta/alpha ratio of a drowsy episode with the preceding and subsequent theta/alpha ratios of wakeful episodes to confirm true drowsy episodes. The analysis revealed varying sensitivities across different channels, with channel F4 showing particularly high sensitivity among six EEG channels. Combining channels according to brain regions notably amplified sensitivity, especially in the frontal region. Additionally, integrating all six channels significantly increased sensitivity. This exploration concluded that sensitivity varies based on the number of EEG channels and their location.

**Fig. 2:**
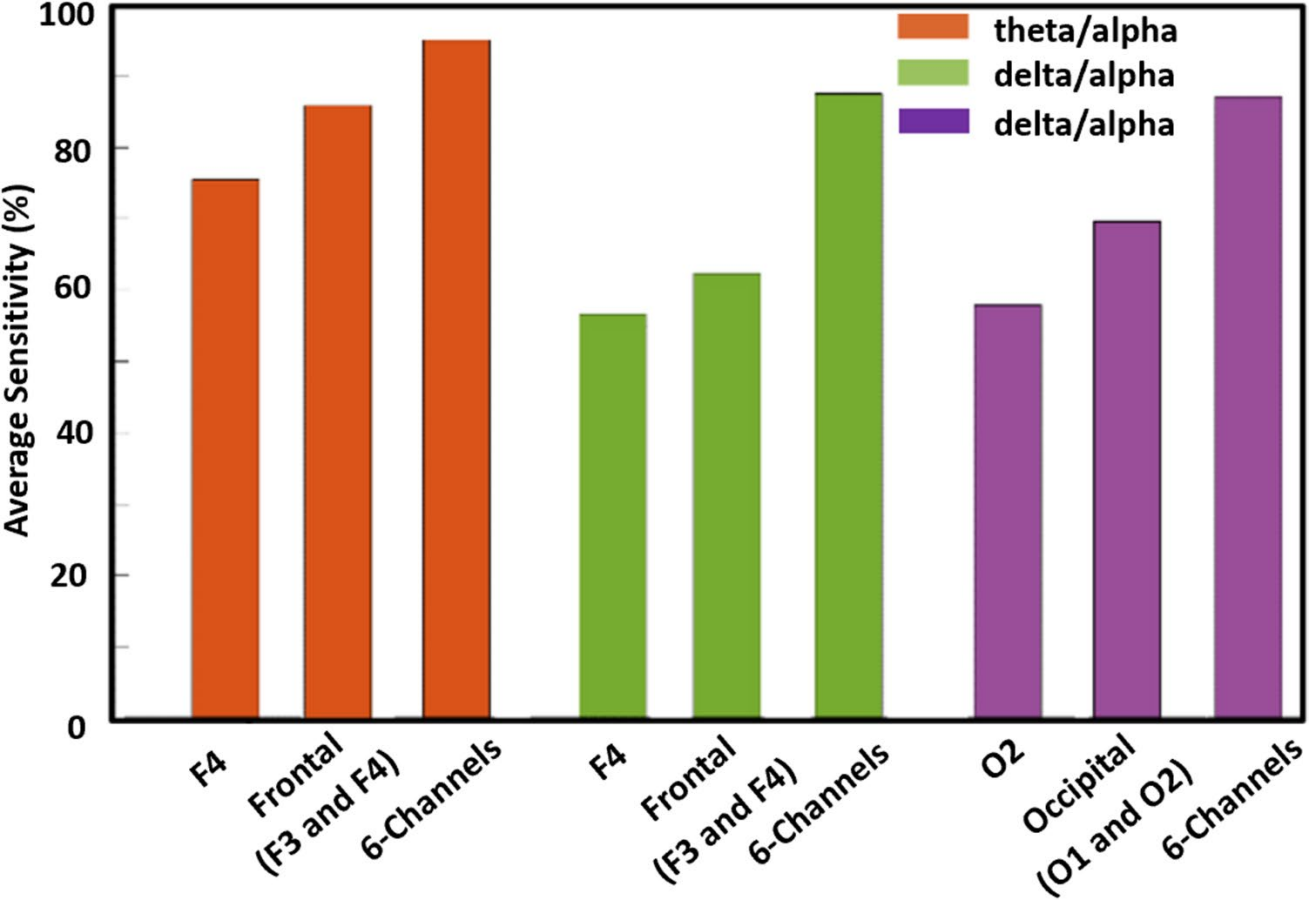
The red, green, and purple bars illustrate that the average sensitivity of matching EEG patterns (theta/alpha ratio or delta/alpha ratio) with visual-based scoring increases with the addition of EEG channels. The red and green bars further indicate that the average sensitivity varies depending on the type of EEG feature used. Additionally, the green and purple bars demonstrate that the average sensitivity is also influenced by the location of the EEG channels

The authors’ subsequent study [18] focused on ten different EEG features instead of a single feature. This approach allowed for the exploration of whether varying the types of features across different channels would enhance sensitivity in detecting variations in EEG patterns related to drowsy and wakeful episodes derived from visual-based scoring. The analysis examined the unique sensitivities of individual EEG features across each channel, with particularly heightened sensitivity in channels F4 and O2 among all six channels. Additionally, it was found that aggregating channels based on brain regions significantly amplified sensitivity. Furthermore, integrating all six channels enhanced sensitivity across all features. These findings confirmed that sensitivity also varies based on the type of EEG feature used, as shown in Fig. 2.

**Fig. 3:**
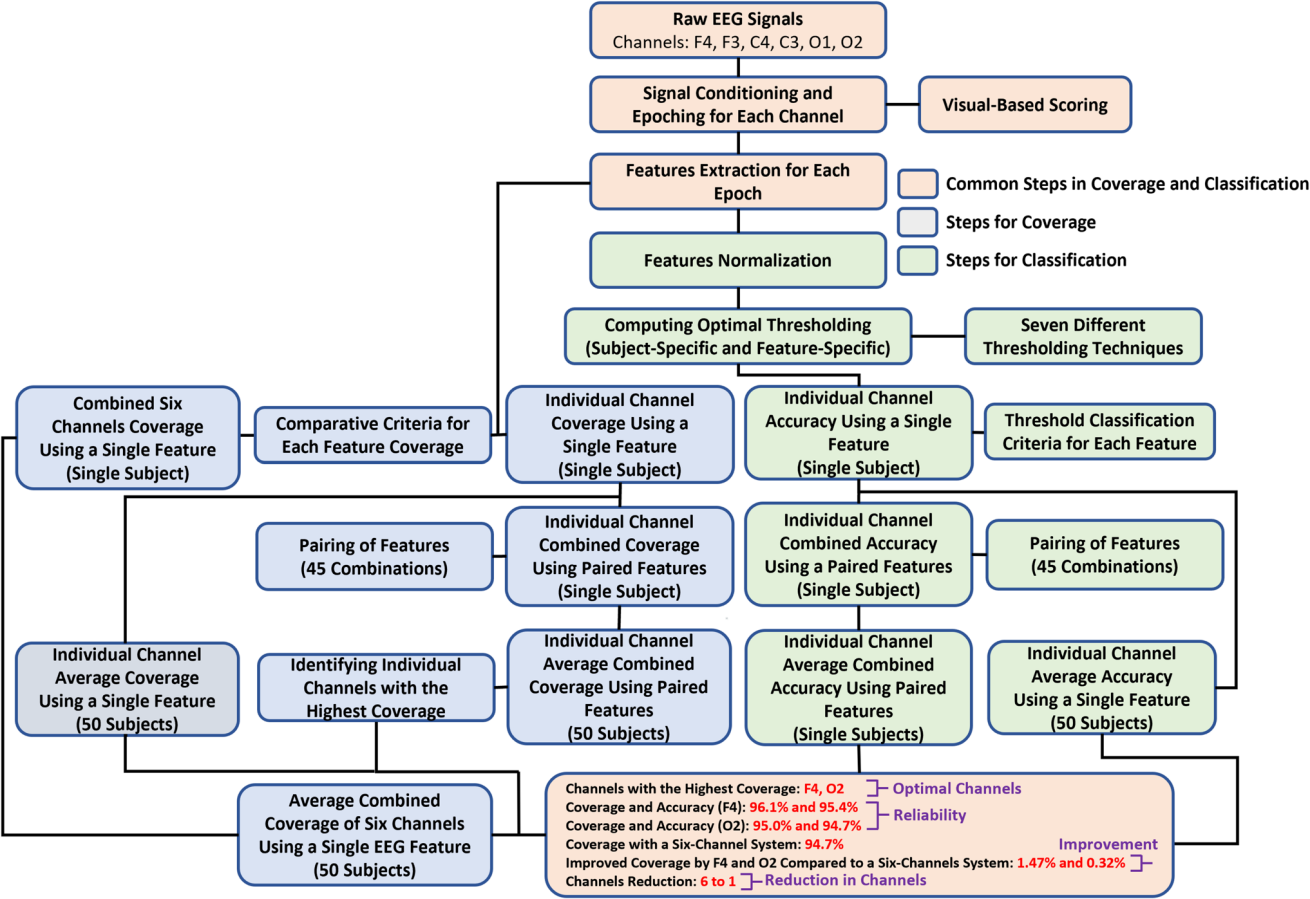
This figure illustrates the algorithm used to first identify the individual EEG channel among all six channels that achieves the highest coverage by optimal pairing of EEG features across fifty subjects and then compare its coverage with a six-channel EEG system, ultimately reducing EEG channels from six to one. Additionally, it focuses on the accuracy of individuals EEG channels in classifying the epochs as wakeful and drowsy based on subject-specific and feature-specific thresholding across fifty subjects

Although increasing the number of EEG channels enhances coverage, it also raises hardware and computational demands. Therefore, the present study aimed to reduce the number of EEG channels to a single optimal EEG channel that could provide coverage equivalent to or exceeding that achieved by combining all channels with a single optimal EEG feature, thus reducing channels from 6 to 1 and computational needs while maintaining efficacy. It was hypothesized that pairing various EEG features, rather than using multiple channels, could achieve this goal. This study not only correlated visual-based scoring with EEG patterns but also employed a classification technique, which could potentially benefit real-time drowsiness detection. Figure 3 presents an overview of the complete process used to validate this premise.

#### Normalization of EEG features

The EEG features extracted for each epoch were normalized to standardize them onto a comparable scale (ranging from 0 to 1), as achieved by Eq. (1). This normalization process aimed to mitigate the substantial discrepancies in raw values observed across different features for each epoch.

1 $${\text{Normalized Feature}}_{(i)}=\frac{{\text{featureData}}_{(i)}- {\text{min}.\text{featureData}}_{(i)}}{{\text{max}.\text{featureData}}_{(i)}- {m\text{in}.\text{featureData}}_{(i)}}$$

The variable “featureData_(i)_” represents the raw data for the feature “i”. The functions min. and max. are employed to determine the minimum and maximum values of “featureData_(i)_”, respectively.

#### Computing subject-specific and feature-specific thresholds

In this work, thresholding classification was employed, in addition to measuring coverage based on comparative criterion, to classify EEG epochs into wakeful and drowsy states for each feature. Determining the optimal subject-specific and feature-specific threshold presented a significant challenge in this methodology, aiming for consistent threshold calculation across fifty subjects and ten features. The objective was to select a threshold technique that could effectively differentiate all wakeful and drowsy episodes, correlating accurately with the labeling (1 or 0) of visual-based scoring. Various thresholding techniques were experimented with to identify the most suitable threshold for each feature and subject, ensuring uniformity in threshold calculation across different EEG features and subjects. The computations of thresholds were performed in MATLAB according to Eqs. 2 through 22, systematically replicated for all features and subjects.

- Threshold based on arithmetic mean (AM)
  2 $${AM}_{\text{wakeful} (i)}=\text{mean} ({\text{normalizedFeatues}}_{\text{i}} (\text{visual}\_\text{scoring}), 1)$$
  3 $${AM}_{\text{drowsy}(i)}=\text{mean} ({\text{normalizedFeatues}}_{\text{i}} (\sim \text{visual}\_\text{scoring}), 1)$$
  4 $${\text{Threshold}}_{AM (i)}=\frac{{AM}_{\text{wakeful} (i)}+ {AM}_{\text{drowsy}(i)}}{2}$$

Here, visual_scoring is an array or vector where each element is either 1 (indicating a wakeful episode) or 0 (indicating a drowsy episode). This array is used to segment the data into wakeful and drowsy categories. AM_wakeful(i)_ is the mean of the normalized features for all epochs labeled as wakeful (where visual_scoring =  = 1), AM_drowsy(i)_ is the mean of the normalized features for all epochs labeled as drowsy (where visual_scoring =  = 0), and Threshold_AM(i)_ is the average of “AM_wakeful(i)_” and “AM_drowsy(i)_”.

- Threshold based on standard deviation (Std)
  5 $${Std}_{\text{wakeful} (i)}=std ({\text{normalizedFeatues}}_{\text{i}} (\text{visual}\_\text{scoring}), 1)$$
  6 $${Std}_{\text{drowsy}(i)}=std ({\text{normalizedFeatues}}_{i} (\sim \text{visual}\_\text{scoring}), 1)$$
  7 $${\text{Threshold}}_{Std (i)}=\frac{{Std}_{\text{wakeful }(i)}+ {Std}_{\text{drowsy}(i)}}{2}$$

Here, Std_wakeful(i)_ is the standard deviation of the normalized features for all epochs labeled as wakeful (where visual_scoring =  = 1), Std_drowsy(i)_ is the standard deviation of the normalized features for all epochs labeled as drowsy (where visual_scoring =  = 0), and Threshold_Std(i)_ is the average of “Std_wakeful(i)_” and “Std_drowsy(i)_”.

- Threshold based on median (M)
  8 $${M}_{\text{wakeful} (i)}=\text{median }({\text{normalizedFeatues}}_{i} (\text{visual}\_\text{scoring}), 1)$$
  9 $${M}_{\text{drowsy}(i)}=\text{median} ({\text{normalizedFeatues}}_{i} (\sim \text{visual}\_\text{scoring}), 1)$$
  10 $${\text{Threshold}}_{M (i)}=\frac{ {M}_{\text{wakeful} (i)}+ {M}_{\text{drowsy} (i)}}{2}$$

Here, M_wakeful(i)_ is the median of the normalized features for all epochs labeled as wakeful (where visual_scoring =  = 1), M_drowsy(i)_ is the median of the normalized features for all epochs labeled as drowsy (where visual_scoring =  = 0), and Threshold_M(i)_ is the average of “M_wakeful(i)_” and “M_drowsy(i)_”.

- Threshold based on trimean (TM)
  11 $${TM}_{\text{wakeful} (i)}=\frac{{Q1}_{\text{wakeful} (i)}+2\times {M}_{\text{wakeful} (i)}+{Q3}_{\text{wakeful} (i)} }{4}$$
  12 $${TM}_{\text{drowsy }(i)}=\frac{{Q1}_{\text{drowsy} (i)}+2\times {M}_{\text{drowsy} (i)}+{Q3}_{\text{drowsy} (i)} }{4}$$
  13 $${\text{Threshold}}_{TM (i)}=\frac{{TM}_{\text{wakeful} (i)}+ {TM}_{\text{drowsy} (i)}}{2}$$

Here, Q1_wakeful(i)_ and Q3_wakeful(i)_ are the first and third quartiles of the wakeful data, while Q1_drowsy(i)_ and Q3_drowsy(i)_ are the first and third quartiles of the drowsy data. The Trimean (TM) combines the median and quartiles to provide a robust measure of central tendency, especially in the presence of outliers. Threshold_TM(i)_ is the average of “TM_wakeful(i)_” and “TM_drowsy(i)_”.

- Threshold based on median absolute deviation (MAD)
  14 $${MAD}_{\text{wakeful} (i)}=\text{mad }({\text{normalizedFeatues}}_{\text{i}} (\text{visual}\_\text{scoring}), 1)$$
  15 $${MAD}_{\text{drowsy}(i)}=\text{mad }({\text{normalizedFeatues}}_{\text{i}} (\sim \text{visual}\_\text{scoring}), 1)$$
  16 $${\text{Threshold}}_{MAD (i)}=\frac{{MAD}_{\text{wakeful} (i)}+ {MAD}_{\text{drowsy}(i)}}{2}$$

Here, MAD is a measure of data spread around the median and it is robust to outliers. MAD_wakeful(i)_ and MAD_drowsy(i)_ represent the MAD of the normalized features for the wakeful and drowsy data, respectively. Threshold_MAD(i)_ is the average of “MAD_wakeful(i)_” and “MAD_drowsy(i)_”.

- Threshold based on interquartile range (IQR)
  17 $${IQR}_{\text{wakeful}(i)}={Q3}_{\text{wakeful }(i)}-{Q1}_{\text{wakeful}(i)}$$
  18 $${IQR}_{\text{drowsy}(i)}={Q3}_{\text{drowsy} (i)}-{Q1}_{\text{drowsy}(i)}$$
  19 $${\text{Threshold}}_{IQR (i)}=\frac{{IQR}_{\text{wakeful}(i)}+ {IQR}_{\text{drowsy}(i)}}{2}$$

Here, IQR is the difference between the third quartile (Q3) and the first quartile (Q1) of a data set, measuring the range within which the central 50% of values fall. IQR_wakeful(i)_ and IQR_drowsy(i)_ represent the IQR for the wakeful and drowsy data, respectively. Threshold_IQR(i)_ is the average of “IQR_wakeful(i)_” and “IQR_drowsy(i)_”.

- Threshold based on robust scaling (RS)
  20 $${RS}_{\text{wakeful} (i)}=\frac{{M}_{\text{wakeful}(i)}- {Q1}_{\text{wakeful}(i)}}{{IQR}_{\text{wakeful}}}$$
  21 $${RS}_{\text{drowsy} (i)}=\frac{{M}_{\text{drowsy}(i)}- {Q1}_{\text{drowsy}(i)}}{{IQR}_{\text{drowsy}}}$$
  22 $${\text{Threshold}}_{RS (i)}=\frac{{RS}_{\text{wakefu}l (i)}+ {RS}_{\text{drowsy} (i)}}{2}$$

Here, RS standardizes data by subtracting the first quartile (Q1) from the median (M) and then dividing by the IQR, normalizing the distribution and reducing the impact of extreme values. RS_wakeful(i)_ and RS_drowsy(i)_ represent the RS for wakeful and drowsy states, respectively. Threshold_RS(i)_ is the average of “RS_wakeful(i)_” and “RS_drowsy(i)_”.

#### Epochs classification based on threshold and coverage assessment using comparative criteria for each feature and subject

“Feature_i_” epochs were classified based on whether the “normalizedFeature_i_” value is above or below a calculated “threshold” for each channel. The decision to classify an epoch as indicating drowsy (or wakeful) was determined by the specific criterion set for each feature, as explained in Table 2, and the accuracy of classification for each channel using individual features was calculated using Eq. 23. Additionally, the study evaluated the coverage of each channel channel (as calculated using Eq. 24), as well as all six channels (as calculated using Eq. 25), using individual features, by the specific criterion set for each feature, as detailed in Table 3.

**Table 2:** This table illustrates the individual criterion for each feature to classify EEG epochs into drowsy or wakeful episodes based on thresholding technique

EEG feature	Criterion for classification based on threshold
Theta/alpha ratio	Here indice “i” shows a feature “theta/alpha ratio” if NormalizedFeatures_i_ > Threshold_i_ Epoch is classified as drowsy else NormalizedFeatures_i_ < Threshold_i_ Epoch is classified as wakeful
Delta/alpha ratio	Criterion is similar to that of “theta/alpha ratio”
Delta/theta ratio	Criterion is similar to that of “theta/alpha ratio”
PSD alpha	Here indice “i” shows a feature “PSD alpha” if NormalizedFeatures_i_ < Thresholdi Epoch is classified as drowsy else NormalizedFeatures_i_ > Thresholdi Epoch is classified as wakeful
PSD theta	Criterion is similar to that of “theta/alpha ratio”
PSD delta	Criterion is similar to that of “theta/alpha ratio”
Spectral entropy	Criterion is similar to that of “PSD alpha”
Spectral spread	Criterion is similar to that of “theta/alpha ratio”
Spectral centroid	Criterion is similar to that of “PSD alpha”
Spectral rolloff	Criterion is similar to that of “PSD alpha”

**Table 3:** This table illustrates the individual criterion for each feature to match the visual-based scoring with the variations in EEG features during the epochs. Here, epoch(i), epoch (i – 1), and epoch (i + 1) correspond drowsy, preceding, and subsequent wakeful episodes derived from visual-based scoring

EEG feature	Novel comparative criterion: comparing drowsy episode value with the values of adjacent wakeful events
Theta/alpha ratio	Here indice “i” shows drowsy episode derived from visual-based scoring If theta_alpha_ratio(i) > theta_alpha_ratio(i—1) && theta_alpha_ratio(i) > theta_alpha_ratio(i + 1) Epoch(i), epoch(i—1), and epoch(i + 1) match with visual-based scoring elseif theta_alpha_ratio(i) > theta_alpha_ratio(i—1) && theta_alpha_ratio(i) < = theta_alpha_ratio(i + 1) Epoch(i + 1) doesn’t match with visual-based scoring elseif theta_alpha_ratio(i) < = theta_alpha_ratio(i—1) && theta_alpha_ratio(i) > theta_alpha_ratio(i + 1) Epoch(i—1) doesn’t match with visual-based scoring else Epoch(i), epoch(i—1), and epoch(i + 1) don’t match with visual-based scoring
Delta/alpha ratio	Criterion is similar to that of ‘theta/alpha ratio’
Delta/theta ratio	Criterion is similar to that of ‘theta/alpha ratio’
PSD alpha	Here indice ‘i’ shows drowsy episode derived from visual-based scoring If PSD_alpha(i) < PSD_alpha(i—1) && PSD_alpha(i) < PSD_alpha(i + 1) Epoch(i), epoch(i—1), and epoch(i + 1) match with visual-based scoring elseif PSD_alpha(i) < PSD_alpha(i—1) && PSD_alpha(i) > = PSD_alpha(i + 1) Epoch(i + 1) doesn’t match with visual-based scoring elseif PSD_alpha(i) > = PSD_alpha(i—1) && PSD_alpha(i) < PSD_alpha(i + 1) Epoch(i—1) doesn’t match with visual-based scoring else Epoch(i), epoch(i −1), and epoch(i + 1) don’t match with visual-based scoring
PSD theta	Criterion is similar to that of ‘theta/alpha ratio’
PSD delta	Criterion is similar to that of ‘theta/alpha ratio’
Spectral entropy	Criterion is similar to that of ‘PSD alpha’
Spectral spread	Criterion is similar to that of ‘theta/alpha ratio’
Spectral spread	Criterion is similar to that of ‘PSD alpha’
Spectral rolloff	Criterion is similar to that of ‘PSD alpha’

23 $$\text{Accuracy of Individual Channel }=\frac{\text{Epochs classified in line with visual}-\text{based scoring }}{\text{Total Number of epochs}}\times 100$$

24 $$\text{Coverage of Individual Channel}=\frac{\text{Epochs matched with visual}-\text{based scoring }}{\text{Total Number of epochs}}\times 100$$

25 $$\text{Combined Coverage of Six Channels}=\left[1-\frac{\text{Epochs not matched by merging six channels}}{\text{Total number of epochs}}\right]\times 100$$

#### Computing the combined coverage and accuracy of paired features for ındividual channels in each subject

A logical OR operation was used to combine the individual classification accuracy and coverage of each feature within a pair (totaling 45 combinations) for each channel and subject. This step aimed to elucidate the relative resemblance between the combined coverage based on comparative criteria and the combined accuracy achieved through threshold technique, ensuring the reliability of the methodology.

- Computing combined classification accuracy

Combined classification accuracy was calculated by assessing whether either of the two features in the pair classified an epoch correctly according to the visual-based scoring. Specifically, for each epoch, if either feature's classification matched the visual scoring, it was considered correct. The accuracy was then determined by averaging these correct classifications across all epochs and expressing it as a percentage.

- Computing combined coverage

To evaluate the combined coverage, we assessed whether at least one feature in each pair accurately matched the EEG patterns during the epochs, as aligned with visual-based scoring. A binary matrix, feature_matrix, was created, where each entry indicates whether a feature's pattern matched (marked as 1) or did not match (marked as 0) the visual scoring. The coverage results of the two features in each pair were then combined using a logical OR operation. The combined coverage was computed as the average of these results across all epochs and expressed as a percentage.

#### Computing average combined coverage and accuracy for each paired features based on ındividual channels across fifty subjects

After computing the combined accuracy and coverage of all possible paired features for all individual channels within each subject, steps from Sections. 2.2.1 to 2.2.4 were replicated across fifty subjects to determine the average combined accuracy and coverage for each channel. This process is summarized in Eqs. 26 and 27. Additionally, the average accuracy and coverage for each channel using individual features across fifty subjects were calculated using Eqs. 28 and 29. Furthermore, the average combined coverage was calculated by integrating all six channels across fifty subjects using Eq. 30.

26 $$\text{Average Combined Accuracy}=\text{mean }\left(\text{combinedAccuracy}\right)$$

27 $$\text{Average Combined Coverage}=\text{mean }\left(\text{combinedCoverage}\right)$$

28 $$\text{Average Accuracy }= \frac{\text{Sum of accuracy of Individual Channel across all subjects }}{\text{Total number of subjects}}$$

29 $$\text{Average Coverage }= \frac{\text{Sum of coverage of Individual Channel across all subjects }}{\text{Total number of subjects}}$$

30 $$\text{Average Combined Covergae }= \frac{\text{Combined coverage of six channels across all subjects }}{\text{Total number of subjects}}$$

#### Identifying the optimal EEG channel by comparing the coverage of ındividual channels to a six-channel system

First, the individual channels with the highest average combined coverage among all six channels analyzed were identified. Next, the coverage of these channels was compared to that obtained by combining all six channels using a single optimal feature to determine the optimal EEG channel. Finally, the top three paired features for each optimal channel were identified, which most effectively classified epochs into drowsy and wakeful episodes, demonstrating heightened coverage consistent with visual-based scoring across 50 subjects.

## Results

### Enhanced average combined coverage and accuracy for ındividual channels focusing on paired features across fifty subjects

**Fig. 4:**
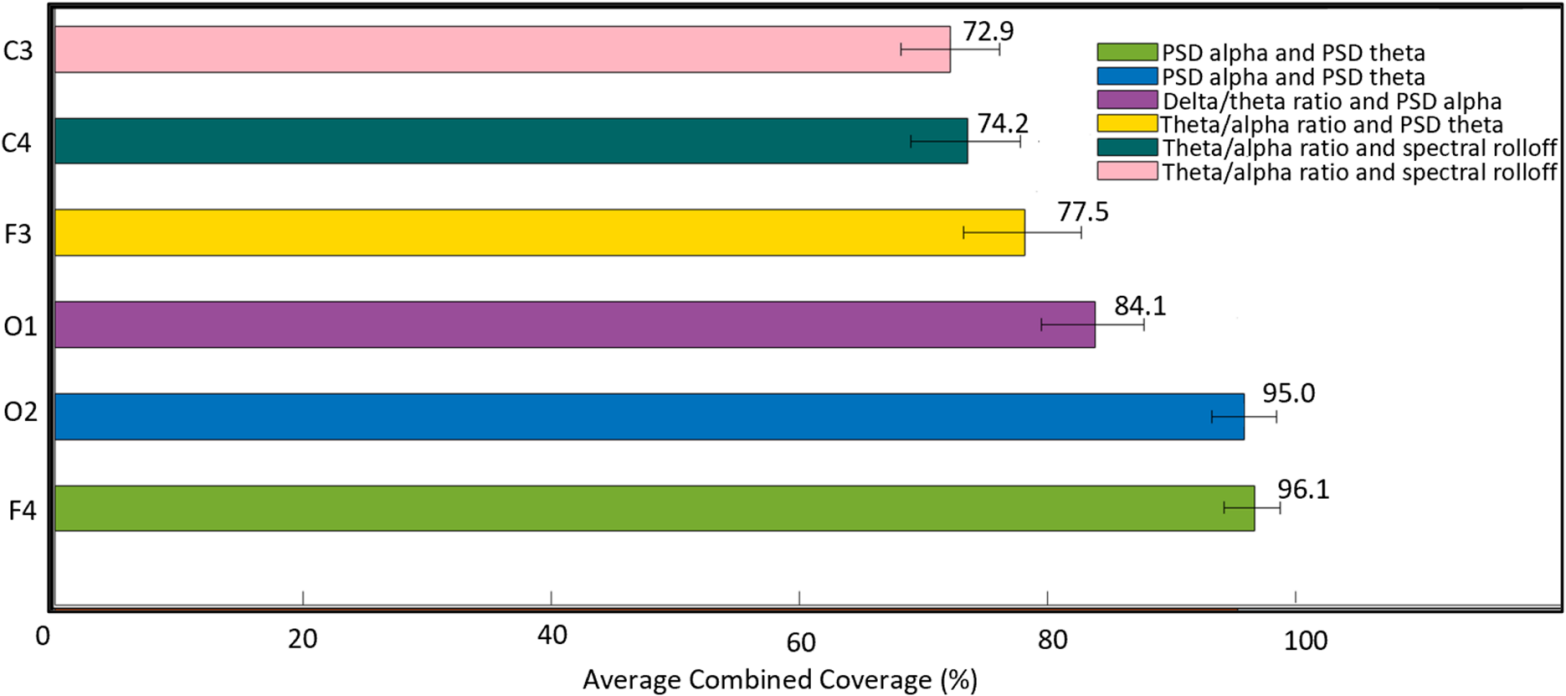
This figure illustrates the average combined coverage for each channel used in this study, determined by the comparative criterion, focusing on optimal paired features selected from 45 possible combinations. Channels F4 and O2 exhibit the highest average combined coverage among all six channels analyzed. Each bar includes a horizontal error line representing the 95% confidence interval (CI), indicating the range within which the true average is likely to fall with 95% certainty

**Fig. 5:**
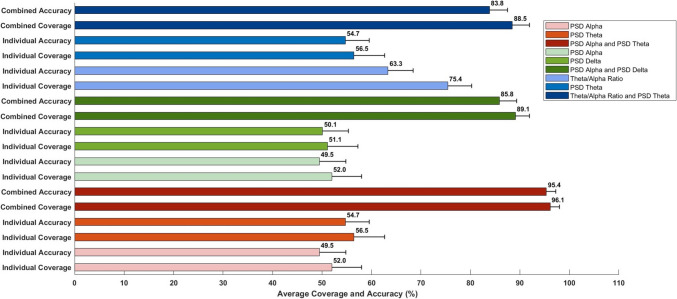
This figure illustrates the average combined accuracy, determined by the median threshold, and the average combined coverage, assessed against comparative criteria, for the top three paired features along with their individual values for channel F4 across fifty subjects. The combination of PSD alpha and PSD theta emerges as the optimal choice, followed by the combinations of PSD alpha and PSD delta, and theta/alpha ratio and PSD theta. Each bar includes a horizontal error line representing the 95% confidence interval (CI), indicating the range within which the true average is likely to fall with 95% certainty

**Fig. 6:**
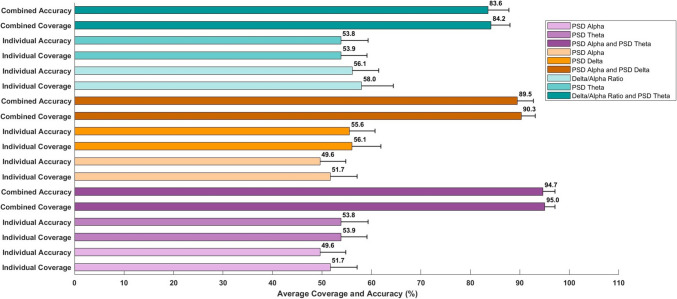
This figure illustrates the average combined accuracy, determined by the median threshold, and the average combined coverage, assessed against comparative criteria, for the top three paired features along with their individual values for channel O2 across fifty subjects. The paired feature combination of PSD alpha and PSD theta emerges as the optimum choice, followed by the combinations of PSD alpha and PSD delta, and delta/alpha ratio and PSD theta. Each bar includes a horizontal error line representing the 95% confidence interval (CI), indicating the range within which the true average is likely to fall with 95% certainty

The average combined coverage was computed using a novel comparative criterion, and the average combined accuracy was determined using thresholding for individual channels across 50 subjects, focusing on paired features. The results, as depicted in Table 4, indicate the enhancement in coverage for individual channels when using optimal paired features compared to using a single optimal feature. Additionally, the average combined coverage varies by channel location, with channels F4 and O2 exhibiting the highest coverage among the six channels analyzed, as shown in Fig. 4, and also varies based on the feature types within each pair. The three optimal feature combinations—those that show heightened coverage for a channel among all 45 possible combinations—are presented for channels F4 and O2 in Figs. 5 and 6. These combinations not only demonstrate elevated average combined coverage and enhanced average combined accuracy but also show variability in these metrics depending on the feature types within each combination.

**Table 4:** This table illustrates the enhancement in coverage for individual channels when using optimal paired features compared to using a single optimal feature

Channels	Coverage of individual channels using optimal single feature (%)	Combined coverage of individual channels using optimal paired feature (%)
F4	75.4	96.1
F3	63.3	77.5
C4	64.0	74.2
C3	62.1	72.9
O1	59.6	84.1
O2	60.1	95.0

Furthermore, to elucidate the impact of feature combination, the average individual coverage and accuracy were assessed, illustrating the enhancements achieved by optimal feature pairing. For channel F4, this improvement is evident in the combination of PSD alpha and PSD theta, where the average combined coverage and accuracy reach 96.1% and 95.4%, respectively, representing a significant enhancement over their individual counterparts, which are 52.0% and 49.5%, respectively, for PSD alpha, and 56.5% and 54.7%, respectively, for PSD theta. For channel O2, a similar improvement was noted in the same combination of PSD alpha and PSD theta, with the average combined coverage and accuracy reaching 95.0% and 94.7%, respectively. This indicates a notable improvement over their corresponding individual values, which are 51.7% and 49.6%, respectively, for PSD alpha, and 53.9% and 53.8%, respectively, for PSD theta. It is notable that across all three combinations and their individual counterparts, the average coverages and accuracies display a remarkable similarity, with the average coverage for each combination and its counterparts slightly higher. This consistent trend persists across all possible feature combinations and their corresponding individual values, thereby bolstering the reliability of the classification methodology. Furthermore, channel F4 exhibited slightly higher values for average combined coverage and average combined accuracy compared to Channel O2.

To assess the generalizability of our classification and coverage analyses for channels F4 and O2, focusing on paired EEG features (PSD alpha and PSD theta) across fifty subjects, we employed Spearman’s rank correlation. This non-parametric method was chosen due to the non-normal distribution of our data, as detailed in Table 5.

**Table 5:** Spearman’s correlations were calculated between episodes derived from visual-based scoring and instances where paired EEG features (PSD alpha and PSD theta) matched these episodes for channels F4 and O2. Similarly, for these channels, correlations were computed between episodes derived from visual-based scoring and instances where epochs classified as wakeful or drowsy aligned with this scoring based on median thresholds, focusing on paired EEG features (PSD alpha and PSD theta). These analyses were conducted across a cohort of fifty subjects

Channel and analysis type	Spearman’s correlation
F4 (Coverage with PSD alpha and theta)	*r* = 0.9838, *p* < 0.001
O2 (Coverage with PSD alpha and theta)	*r* = 0.9764, *p* < 0.001
F4 (Accuracy with PSD alpha and theta)	*r* = 0.9798, *p* < 0.001
O2 (Accuracy with PSD alpha and theta)	*r* = 0.9718, *p* < 0.001

### Identifying optimal EEG channels: comparing coverage of ındividual channels with optimal paired features to a multichannel system using a single optimal feature

**Fig. 7:**
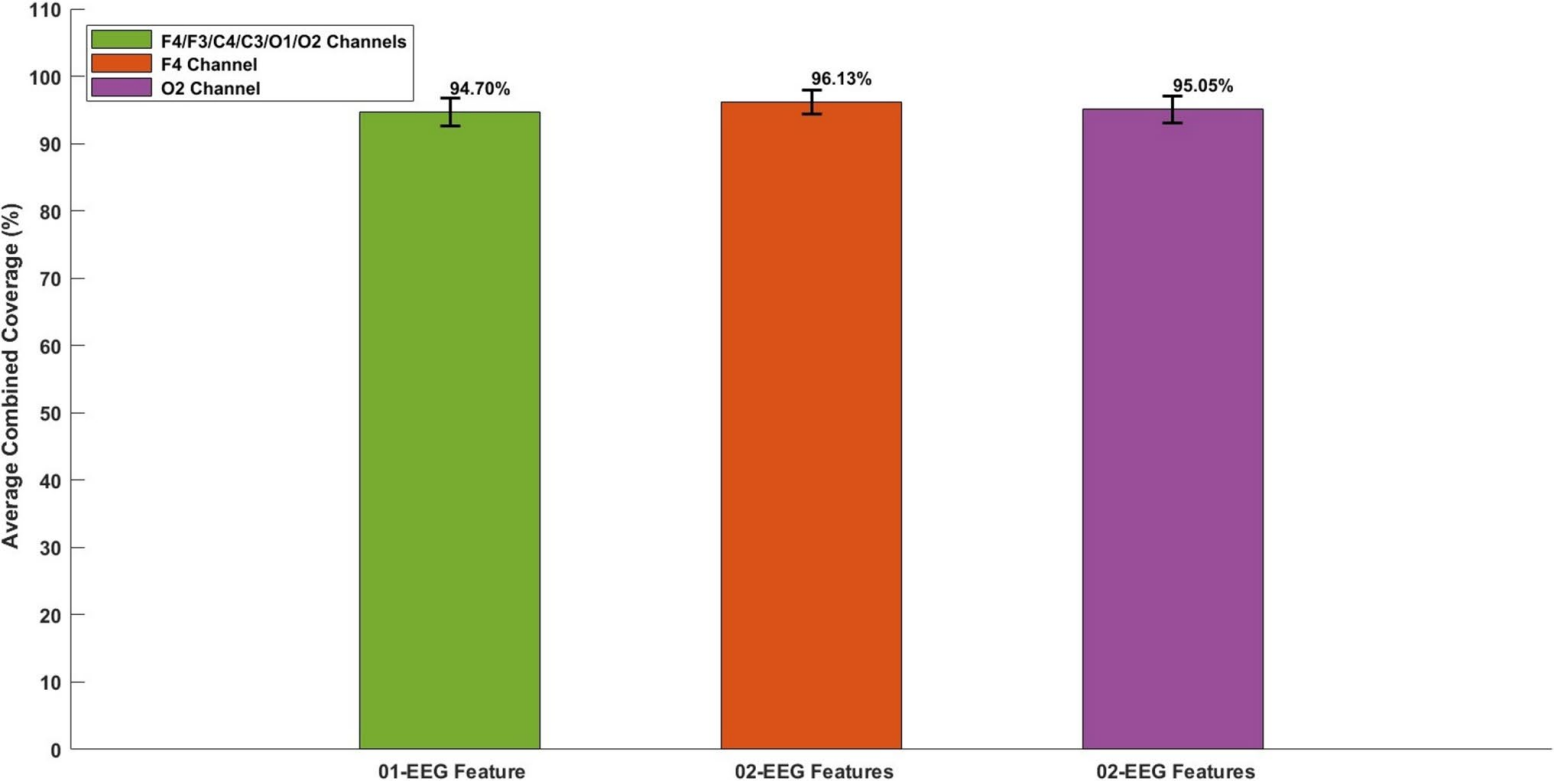
This figure clearly shows that the average combined coverage of individual channels F4 and O2, achieved by combining two optimal features out of ten, exceeds the average combined coverage obtained by integrating six channels using one optimal feature. Each bar includes a horizontal error line representing the 95% confidence interval (CI), indicating the range within which the true average is likely to fall with 95% certainty

It was found that by optimally pairing EEG features such as PSD alpha and PSD theta, channels F4 and O2 achieved heightened average combined coverage—96.1% for F4 and 95.0% for O2—compared to the 94.7% average combined coverage obtained by considering all six channels with a single optimal feature (theta/alpha ratio), as shown in Fig. 7. These channels were termed as the optimal channels. This optimal pairing allows for a reduction in the number of EEG channels from 6 to 1 for the same purpose, with slightly greater efficacy—1.47% for channel F4 and 0.32% for channel O2.

### Variation in average combined accuracy by altering thresholding techniques

**Fig. 8:**
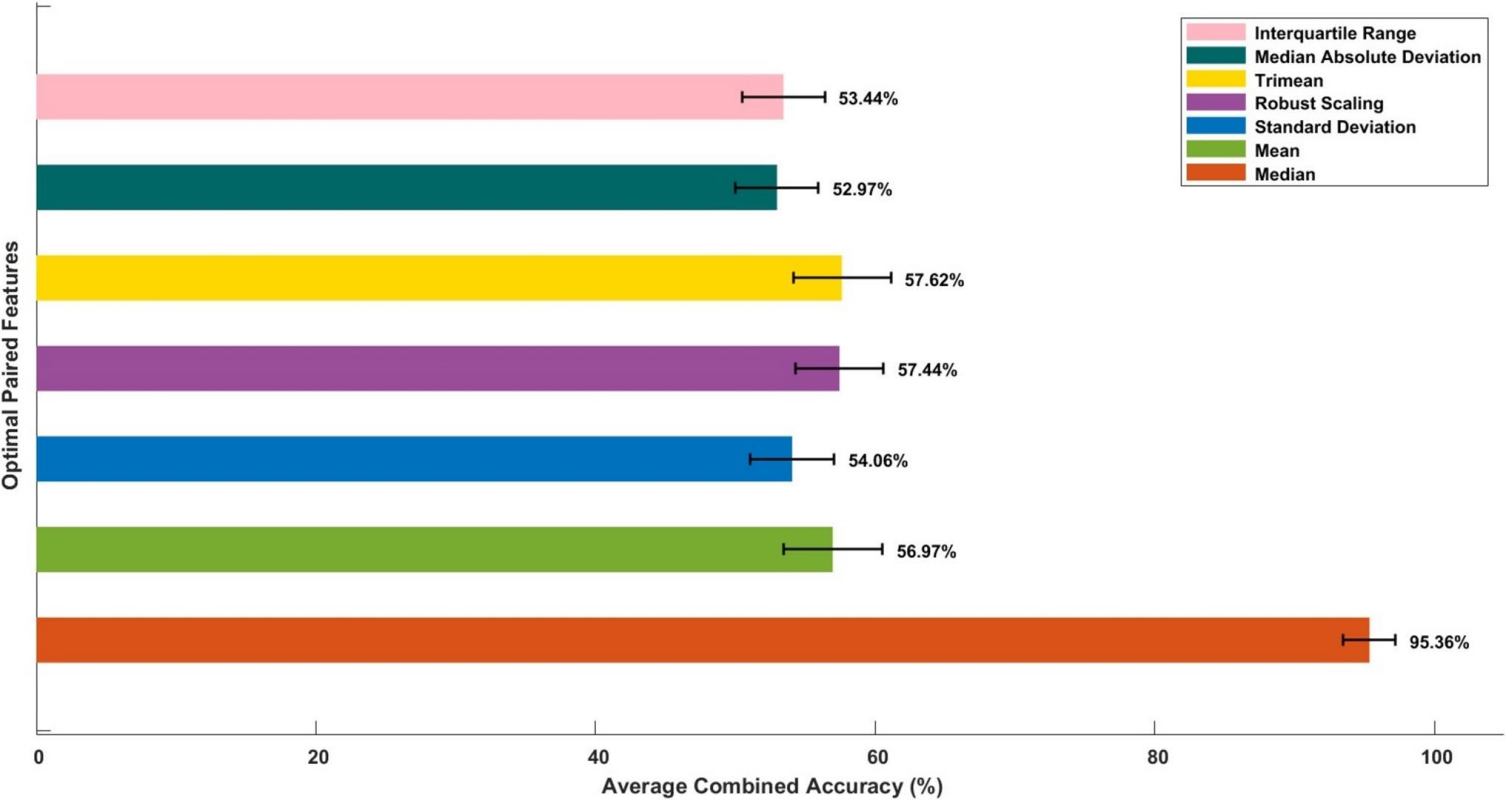
This figure clearly demonstrates that the average combined accuracy varies depending on the threshold computation technique. Specifically, PSD alpha and PSD theta were identified as the optimal paired features for median threshold, while theta/alpha ratio and spectral spread emerged as the optimal paired features for the remaining threshold techniques. Each bar includes a horizontal error line representing the 95% confidence interval (CI), indicating the range within which the true average is likely to fall with 95% certainty

Thresholds were calculated for each feature, and it was noted that their values vary not only across features within a subject but also across different subjects. Additionally, variations in average combined accuracy and their individual counterparts were evaluated with changes in the threshold computing technique. It was found that employing threshold calculation based on the median resulted in heightened average combined accuracy (95.4%) for PSD alpha and PSD theta compared to other techniques, as illustrated in Fig. 8.

## Discussion

Although a multi-channel EEG system provides extensive coverage for drowsiness detection, it is less wearable-friendly due to its computational demands, hardware requirements, and user discomfort. Conversely, a single-channel EEG system is more user-friendly, cost-effective, and quicker to set up, although it sacrifices some coverage [20]. Given the real-world constraints of wearable drowsiness detection devices, the present study aimed to reduce the number of EEG channels to a single optimal channel that could provide coverage equivalent to or exceeding that of a multi-channel system using a single optimal EEG feature. It was hypothesized that pairing optimal EEG features within a single channel, rather than using multiple channels with a single optimal feature, could achieve this goal. Additionally, to enhance the reliability of the methodology, each EEG epoch was classified as wakeful or drowsy by comparing the normalized value of the EEG feature to an optimal threshold value, ensuring consistency with visual-based scoring.

Previous studies that employed minimal EEG channels (typically up to two) for drowsiness detection selected channel locations based on neurophysiological knowledge [21–28]. However, these studies were limited by their focus on one or two channels targeting a specific brain region without exploring other channels or regions for potential improvements in detection coverage. Studies [18, 32] have shown that sensitivity in identifying EEG patterns associated with drowsy and wakeful states varies with the number of channels, their spatial locations, and the EEG features used. Moreover, previous research lacked a methodology for reducing the number of EEG channels by identifying the optimal EEG channel with equal or higher coverage compared to a multichannel system. In contrast, the present study utilized six EEG channels (F3, F4, C3, C4, O1, and O2), selected based on established literature linking EEG modifications in occipital (particularly O2) and central regions to driver drowsiness [43, 44]. Notably, the frontal region, particularly F4, has been identified as highly sensitive to changes in the theta and alpha frequency bands during transitions from wakefulness to drowsiness [32, 45]. By employing these channels, the present study effectively captured neural activity patterns associated with drowsy and wakeful episodes while driving. The effectiveness of using these channels is demonstrated by the results which achieved up to 94.7% coverage when combining all six channels. Additionally, the systematic approach to identify the optimal EEG channel among all six channels revealed that a single optimal channel (F4), using paired features (PSD alpha and PSD theta), could achieve 96.1% coverage— slightly surpassing the coverage obtained with the six-channel system.

Among the ten selected EEG features, three were power spectral density (PSD) measures, three were ratios of these measures, and four were spectral characteristics. These features were chosen for their significant variation during the transition from wakefulness to drowsiness. Several studies have observed heightened theta [46] and delta [46, 47] brain activity as drivers transition from wakefulness to drowsiness. Other research has indicated that drowsiness is often associated with decreased EEG activity, characterized by an increased dominance of the theta frequency band [48, 49] or a decline in alpha activity, particularly when the eyes are closed [50]. The authors’ previous study [32] observed an increase in the theta/alpha ratio during microsleep episodes and a decrease during wakefulness. Similarly, another authors’ study [18] identified an increase in the delta/alpha ratio, delta/theta ratio, and spectral spread, but a decrease in spectral centroid and spectral rolloff as drivers shifted from wakefulness to drowsiness. Additionally, Sriraam N et al. [51] and Krishnan P et al. [52] found higher mean spectral entropy values during wakefulness compared to periods of increased sleepiness. Building on these findings, the present study established feature dependent criteria for classifying EEG epochs as wakeful or drowsy. For example, if the normalized value of the theta/alpha ratio associated with an epoch exceeds the optimal threshold value, the epoch is classified as drowsy; conversely, it is classified as wakeful if the ratio is lower. Similarly, the study developed feature dependent comparative criteria for identifying coverage. For instance, if the theta/alpha ratio of an epoch linked to a potential drowsy episode—based on visual scoring—is greater than that of the preceding and subsequent epochs corresponding to potential wakeful episodes, then this epoch aligns with the visual-based scoring. Conversely, if its value is lower than that of both neighboring epochs, then this epoch does not match the visual-based scoring.

Although recent advancements in machine learning and deep learning have shown promise in accurately classifying EEG signals [53, 54], these methods were deliberately excluded from the research for specific reasons. The EEG data were segmented based on visual-based scoring. Given the inherent variability in the duration of these episodes both within and across subjects, EEG segment lengths were not consistent. Consequently, employing a fixed-length window for EEG segmentation [26–28], which is common in deep learning techniques, proved to be unsuitable [55]. To address the diverse lengths of EEG segments and maintain accurate, subject-specific classification, a threshold-based classification approach was adopted. After evaluating various thresholding methods, it was found that median thresholding yielded higher classification accuracy, a result consistent across all subjects. Additionally, it was observed that the threshold value varied across features within a subject, and also differed from one subject to another.

The present study identified channels F4 and O2 as the optimal EEG channels among the six channels analyzed, based on their improved coverage when utilizing optimal paired EEG features, surpassing the coverage provided by all six channels combined. Although previous studies [43–45] also concluded that F4 and O2 are more sensitive in detecting EEG patterns associated with drowsy and wakeful states, they lacked quantification of these sensitivities. Additionally, the study identified PSD alpha and PSD theta as the optimal paired EEG features among 45 combinations, consistent with previous studies [46][48–50], which observed variations in the theta and alpha frequency bands during transitions from wakeful to drowsy states.

The methodology achieved successful classification of 884 out of 927 epochs consistent with visual-based scoring for channel F4, slightly better than channel O2, with 448 (94.5%) classified as wakeful out of 474 and 436 (96.2%) classified as drowsy out of 453. This level of accuracy surpasses those reported in studies 82.8% [21], 90.4 [22], 81% [23], 91.9% [24], 72.7% [25], 91% [26], 85.5 [27], and 88.8 [28]. Additionally, the coverage of each channel was assessed using a novel comparative criterion, yielding results consistent with the classification outcomes, thereby further validating the reliability of the methodology.

### Limitations of the study and future perspective

The present study employed a driving simulator designed for male bus drivers, focusing on those diagnosed with OSA, given its higher prevalence among males, especially those operating heavy vehicles [56]. While the study was restricted to traditional features and employed seven techniques for finding the optimum thresholding method, exploring non-linear features and alternative thresholding techniques could potentially enhance classification accuracy. To avoid the labor-intensive and error-prone process of manual EEG scoring, it was deliberately excluded from the methodology. For every new participant, a subject-specific threshold needs to be calibrated before starting the real-time drive. Although the study was limited to six EEG channels based on established research, exploring additional channels and brain regions could enhance detection coverage. Future research should apply this methodology to real-time classification to evaluate its efficacy in practical settings.

## Conclusion

The study successfully reduced the number of EEG channels from six to one by identifying an optimal channel (F4 or O2) using an optimal paired features, providing coverage equal to or better than that of a six-channel system using a single optimal feature. The subject-specific, feature-specific classification achieved higher accuracy in distinguishing EEG epochs as drowsy or wakeful, consistent with visual-based scoring, using the optimal channel. It was also found that channel F4 offers slightly better coverage than channel O2. Additionally, the study identified an optimal pair of EEG features (PSD alpha and PSD theta) that align well with visual-based scoring. Through evaluating various thresholding techniques, it was found that median-based thresholding consistently produced higher accuracy across all subjects.

The study demonstrates that an optimal EEG channel can achieve coverage comparable to that of a multi-channel system. This finding suggests potential reductions in hardware and computational demands for wearable drowsiness detection devices.
